# Hierarchical coassembly of DNA–triptycene hybrid molecular building blocks and zinc protoporphyrin IX

**DOI:** 10.3762/bjnano.7.62

**Published:** 2016-05-12

**Authors:** Rina Kumari, Sumit Singh, Mohan Monisha, Sourav Bhowmick, Anindya Roy, Neeladri Das, Prolay Das

**Affiliations:** 1 Department of Chemistry, IIT Patna, Bihata 801118, India; 2 Department of Biotechnology, IIT Hyderabad, Hyderabad, 502205, India

**Keywords:** DNA nanostructure, DNA–organic hybrid, DNA self-assembly, 2,6,14-triptycenetripropiolic acid, zinc protoporphyrin IX

## Abstract

Herein, we describe the successful construction of composite DNA nanostructures by the self-assembly of complementary symmetrical 2,6,14-triptycenetripropiolic acid (TPA)–DNA building blocks and zinc protoporphyrin IX (Zn PpIX). DNA–organic molecule scaffolds for the composite DNA nanostructure were constructed through covalent conjugation of TPA with 5′-C12-amine-terminated modified single strand DNA (ssDNA) and its complementary strand. The repeated covalent conjugation of TPA with DNA was confirmed by using denaturing polyacrylamide gel electrophoresis (PAGE), reverse-phase high-performance liquid chromatography (RP-HPLC) and matrix-assisted laser desorption/ionization time-of-flight (MALDI-TOF). The biologically relevant photosensitizer Zn PpIX was used to direct the hybridization-mediated self-assembly of DNA–TPA molecular building blocks as well as a model guest molecule within the DNA–TPA supramolecular self-assembly. The formation of fiber-like composite DNA nanostructures was observed. Native PAGE, circular dichroism (CD) and atomic force microscopy (AFM) have been utilized for analyzing the formation of DNA nanofibers after the coassembly. Computational methods were applied to discern the theoretical dimension of the DNA–TPA molecular building block of the nanofibers. A notable change in photocatalytic efficiency of Zn PpIX was observed when it was inside the TPA–DNA scaffold. The significant increase in ROS generation by Zn PpIX when trapped in this biocompatible DNA–TPA hybrid nanofiber may be an effective tool to explore photodynamic therapy (PDT) applications as well as photocatalytic reactions.

## Introduction

Hybrid nanomaterials resulting from the covalent conjugation of DNA with organic molecules [1–10], polymers [11], metal complexes [12–13], and nanoparticles [14] have recently attracted substantial attention. These have potential applications in DNA detection [15–17], molecular electronics [18–20], catalysis [21], and drug delivery [22–23]. For the creation of DNA–organic hybrid molecular building blocks, the selection of organic molecules and their inherent directionality have been found to be the most important determinant for the desired system to have improved functional properties and stability [1]. Reportedly, several nanostructures have been developed by conjugating planar organic molecules with DNA. The inherent planarity and symmetry in these molecules yielded 2D structures [24–25]. Recently, DNA–organic hybrids having a definite angular directionality have yielded interesting nanostructure with 3D topology [26–27]. Herein, for the first time we report the construction and subsequent self-assembly of DNA–organic hybrid using triptycene as the organic molecule that allows for the definite disposition of the DNA strands in three dimensions.

Triptycene is an interesting molecule having *D*_3_*_h_* symmetry with Y-shaped structure. It has attracted considerable attention in nanotechnology due some of its unique physical and chemical properties [28–29]. Materials derived from triptycene usually exhibit a large surface area with high pore volumes due to the internal free volume (IFV) of the triptycene skeleton. It provides a rigid contortion site for polymers, restricts the efficient packing and promotes spatial separation of polymer backbones [30–31]. A great challenge remains in the design of triptycence-based complex functional systems having a long-range alignment of molecules over different scales in a hierarchically organized manner in aqueous media. This limitation could be overcome by functionalization of triptycene molecules with materials having excellent water solubility and functional properties such as DNA. The non-covalent interaction of triptycene derivatives with DNA has been investigated [32–35]. However, the covalent conjugation of triptycene derivatives with any biomolecules has not yet been reported. Using the functionalization of tryptycene with DNA, the construction of tailorable porous structures is envisioned here.

The insertion of synthetic molecules into DNA could alter the assembly outcome as well as the orientation of the DNA strands relative to one another in a programmed manner [21,36–39]. The diverse structural features and functionalities of the organic core such as luminescence, redox, magnetic, and catalytic properties play a vital role in enhancing the versatility of the discrete well-defined DNA nanostructures [40]. Additionally; these DNA–organic hybrids are endowed with better base pairing fidelity, stability, DNA economy and others [41–42]. Supramolecular structures having a confined space can accommodate small molecules that are suitable for catalysis and other applications [43–44].These small molecules also provide the template for the construction of self-assembled supramolecular structures that undergo several self-correction steps in the due course of the construction of their complex structures [45]. These structures may be nanopores, nanofibers, nanotubes and polymeric networks [46–47].

**Scheme 1:**
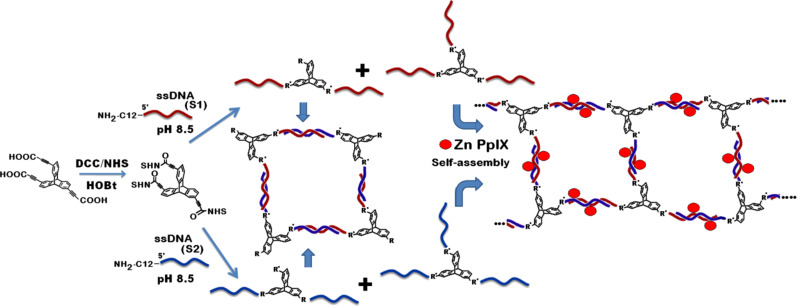
Schematic representation of creation of nanostructures from DNA–TPA hybrid self-assembly. The number and location of Zn PpIX molecules in the scheme are symbolic.

In this study, we report the synthesis of the DNA–TPA scaffolds by covalent conjugation of 2,6,14-triptycenetripropiolic acid (TPA) with amine-modified 12-mer ssDNA and coassembly with zinc protoporphyrin IX(Zn PpIX) to form composite DNA nanostructures (Scheme 1). Porphyrins are biologically highly relevant molecules and their biocompatibility is notable. Porphyrin derivatives are widely used as photosensitizers in PDT to produce reactive oxygen species (ROS). Reportedly, Zn PpIX can interact with dsDNA in “outside stacking mode”. Therefore, the rationale to use Zn PpIX in the present study is two-fold. Firstly, in the interaction of Zn PpIX with DNA, the former is hypothesized to provide template for assembly formation [45]. Secondly, the generation of ROS through excitation of porphyrins is an established fact [48].

The covalent conjugation of TPA with ssDNA was characterized by using denaturing polyacrylamide gel electrophoresis (PAGE), reversed-phase high-performance liquid chromatography (RP-HPLC) and matrix-assisted laser desorption/ionization time-of-flight (MALDI-TOF) analysis. The assembly of DNA–TPA in the presence and absence of Zn PpIX was characterized by native PAGE, circular dichroism (CD), thermal melting analysis, mung bean nuclease digestion (MBN), computational studies and atomic force microscopy (AFM). These comprehensive experimental and computational studies provided detailed information pertaining to the formation of composite DNA nanostructures. We also report excellent photocatalytic activity of these composite nanostructures wherein the oxidation of dihydrorhodamine 123 (DHR 123) into rhodamine 123 (R 123) under UV irradiation has been studied in aqueous environment. Furthermore, these composites exhibit higher catalytic activity with regard to the light-induced oxidation of DHR 123 than the corresponding free Zn PpIX due to enhanced local confinement of ROS in the composite. Therefore, considering this feature, this system could be explored further for PDT, photodynamic antimicrobial chemotherapy (PACT) and catalysis applications. Our work also provides insight for triptycene-like molecules containing internal free volume (IFV) to be used as a functional molecule for construction of composite DNA nanostructures. These composite nanostructures can be important in biology and as promising materials in nanotechnology, e.g., in building smart drug carriers, sensors or materials with significant property combinations.

## Experimental

### General

HPLC-purified single strand 12-mer 5′-(CH_2_)_12_-amine-modified DNA, 1,3-dicyclohexylcarbodiimide (DCC), *N*-hydroxysuccinimide (NHS), hydroxybenzotriazole (HOBt), dimethylformamide (DMF), acetonitrile (CH_3_CN), triethylammonium acetate (TEAA), Pp IX, zinc acetate, acrylamide, bis(acrylamide), and all chemicals required for buffer preparation and gel electrophoresis were obtained from either Sigma-Aldrich or Alfa Aesar and used without further purification. The sequences of ssDNA used for conjugation with TPA and subsequent self-assembly are given in Table 1.

**Table 1:** Sequences of ssDNA used for conjugation with TPA and subsequent self-assembly.

	sequence

S1	5′-[(CH_2_)_12_-NH_2_]-TCA GTC AAC AGC-3’
S2	5′-[(CH_2_)_12_-NH_2_]-GCT GTT GAC TGA-3’

### Syntheses and characterizations

#### Synthesis and characterization of triptycene derivatives

2,6,14-Triptycenetripropiolic acid was prepared according to the reported literature procedure [35]. The compound was characterized by using NMR, and mass and elemental analyses.

#### Conjugation of amine-modified ssDNA with succinimidyl-activated TPA ester

HPLC-purified single strand 12-mer 5′-amine-modified DNA was conjugated with succinimidyl-activated TPA ester through amide coupling in solution. The two complementary sequences of DNA S1 and S2 were conjugated separately with TPA. 6 µL (3 nmol) amine-modified ssDNA was mixed with 0.5 µL (0.8 nm) activated ester in sodium bicarbonate buffer (0.1 M) at pH 8.5. The mixture was heated to 55 °C for 18 h followed by continuous vortexing. The reaction mixture was dialyzed using a dialysis membrane (MWCO 1 kDa) in suitable dialysis buffer to exclude small molecule impurities. Denaturing PAGE (20%) was used for visualization and purification of the DNA–TPA conjugates. The desired bands were excised from the PAGE and the DNA–TPA conjugates were purified using extraction buffer and ethanol washing. These purified conjugates were used for subsequent characterization and further downstream experiments.

#### Self-assembly of DNA–TPA hybrid units

Equimolar ratios of S1 and S2 DNA–TPA units were hybridized after PAGE purification. The hybridization was performed by annealing S1 DNA–TPA with complementary S2 DNA-TPA conjugates in the absence and presence Zn PpIX (2 nmol/nmol of dsDNA) in 10 mM sodium phosphate buffer (NaPi), 10 mM magnesium chloride and 100 mM sodium chloride. The samples were first heated to 90 °C and then slowly cooled to 20 °C with a ramp of 0.1 °C/s and then stored at 4 °C. For all experiments that involve Zn–protoporphyrin (Zn PpIX), the compound was added during the annealing process of the DNA conjugates at a temperature of 60 °C.

#### Thermal melting

The self-assembly of DNA–TPA hybrid structures was studied by optical melting experiments using a Peltier controlled UV–vis spectrophotometer (Bioquest, Cecil, UK). Equimolar ratios of S1 DNA-TPA with S2 DNA-TPA were annealed in 50 µL of total volume of 10 mM sodium phosphate buffer, 10 mM magnesium chloride and 100 mM NaCl (pH 7.2) by heating to 90 °C and allowing the solution to cool slowly to 4 °C at a rate of 0.1 °C per minute over 4 h. Zn PpIX was added during assembly. Hybridized mixtures were denatured by heating the annealed samples from 20 to 90 °C while monitoring the UV absorbance at 260 nm to observe the melting progress. The temperature inside the cuvette was determined with a platinum probe. The absorbance data were analyzed to obtain the melting temperature (*T*_m_) of the samples.

#### Native PAGE to detect self-assembly and MBN activity

The formation of self-assembled products from DNA–TPA conjugates were observed in native PAGE after hybridization. The hybridized products were characterized by native PAGE at 25 °C for 1 h at 200 V and stained with SYBR^®^ Gold. The image was captured by an UVP-Gel documentation system.

#### Dynamic light scattering (DLS)

The size distributions of the DNA nanoconstructs in aqueous solution were obtained by CONTIN analysis of the DLS (Delsa Nano C Particle Analyser, Beckman-Coulter) data. The measurements have been carried out in 100 µL annealing buffer (10 mM sodium phosphate, 75 mM NaCl and 5 mM MgCl_2_) at pH 7.5. The samples were transferred into microcuvettes (Hellma cell) and kept at 20 °C for 2 min prior to measurement. At least five measurements were performed for each sample at 20 °C at a scattering angle of 165°. The data acquisition time for each measurement was 1 h. All buffer solutions were filtered through syringe filters prior to use to remove dust particles.

#### Photocatalytic activity of composite nanostructure

The photocatalytic efficiency of composite DNA nanostructure was evaluated by monitoring the oxidation of DHR 123 (non-fluorescent) into R 123 (fluorescent) by reactive oxygen species (ROS) generated from Zn PpIX under UV light irradiation. In this study, 1 nmol of DHR 123 was added to 2 mL aqueous solution of Zn PpIX (10 µM, 20 nmol) mixed in dark and irradiated with a UV lamp. The efficiency of conversion from DHR 123 to R 123 was analyzed by UV–vis and steady-state fluorescence spectroscopy, before and after irradiation of samples upon addition of DHR 123 at an excitation wavelength of 485 nm and an emission wavelength of 528 nm. The effective concentration of Zn PpIX was taken constant for all the measurements. The percentage of enhancement in degree of oxidation (% EDO) of DHR 123 in the presence of DNA–TPA Zn PpIX nanofiber at λ_max_ = 500 nm and λ_em_ = 534 nm was determined by using the equation as follows:


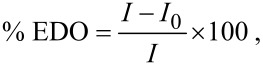


where *I*_0_ is the absorption or the fluorescent intensity of oxidized DHR 123 by free Zn PpIX, and *I* is the fluorescent or absorption intensity in the presence of DNA–Zn PpIX and DNA–Zn PpIX nanofiber upon 2 min UV irradiation.

#### Computational study

In order to resolve the formation of higher ordered structures from assemblies of triconjugated DNA–TPA system, we conducted computational studies on the smallest possible structures generated before the formation of higher-order structures during assemblies. We sketched their 3D structure by using ChemDraw, Maestro’s Build panel and Schrödinger, LLC, New York, NY, 2014 softwares.

## Results and Discussion

### Synthesis and characterization of 2,6,14-triptycenetripropiolic acid

**Scheme 2:**
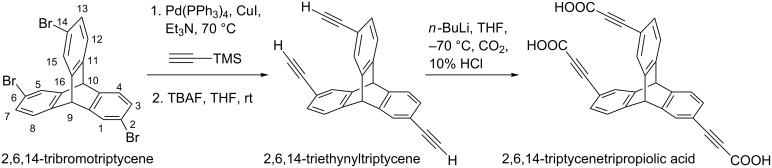
Synthesis of 2,6,14-triptycenetripropiolic acid.

2,6,14-triptycenetripropiolicacid (TPA) was synthesized from 2,6,14-tribromotriptycene in three steps as outlined in Scheme 2. The purity of the compound was confirmed by using NMR, mass spectrometry and elemental analysis [35].

### Synthesis and characterization of DNA–TPA hybrid building blocks

**Figure 1:**
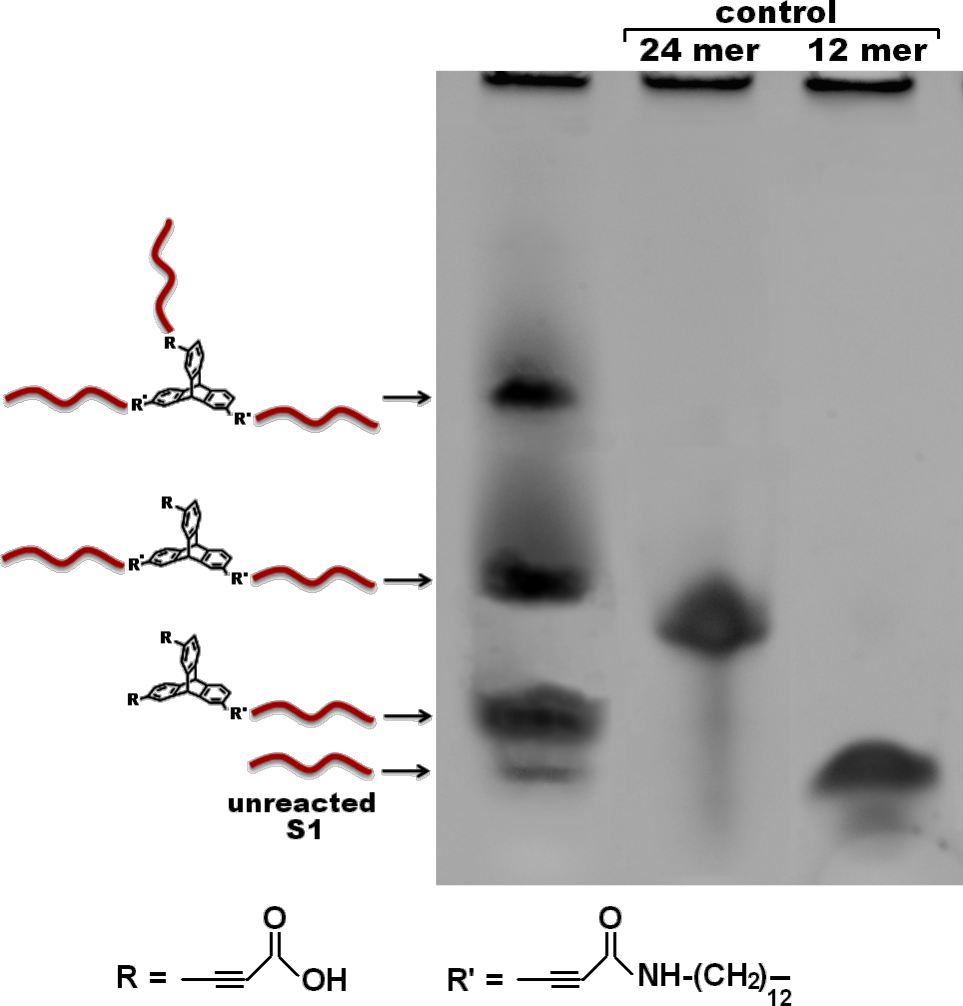
20% denaturing PAGE analysis of DNA (S1)–TPA conjugates showing a decrease in gel mobility of the conjugates upon successive conjugation of ssDNA strands to the triptycene core.

The covalent conjugation of TPA with ssDNA is reported here for the first time. We optimized the DCC/NHS-mediated cross coupling reaction to covalently attach the carboxylic acid group of TPA with the amine functionality of modified ssDNA. The covalent conjugation proceeds through the formation of a 2,6,14-TPA–succinimidyl ester intermediate. A catalytic amount of hydroxybenzotriazole (HOBt) was used as additive for the facile formation of the activated ester [49]. A calculated amount of ssDNA (S1) was added to the activated ester in the second step. An excess of 5′-amine-modified S1 was used to maximize the formation of the DNA–TPA triconjugates. The crude reaction mixtures were purified by dialysis (MWCO 1 kDa) to eliminate the salts and small molecule impurities. The products of the solution-phase amide cross coupling between TPA and DNA was resolved with denaturing PAGE (Figure 1) and further characterized by RP-HPLC and MALDI-TOF mass spectrometry (MS).

Four bands were observed with 20% denaturing PAGE following the conjugation of ssDNA with TPA. With the availability of three carboxylic acid groups in TPA, three products are possible corresponding to the number of ssDNA that could be attached to a single TPA molecule. The formation of mono- (S1-TPA), di- ((S1)_2_-TPA)) and triconjugated ((S1)_3_-TPA)) hybrids was observed with PAGE. The mobility of the three species is distinctly different, which opens a way for their purification with PAGE and subsequent characterization. The yield of the triconjugated species was found to be lower than the mono- and diconjugated counterparts. The gel mobility of the DNA–TPA hybrids is lower than that of normal ssDNA (12 and 24 bases long) units that were used as controls due to the presence of the TPA molecule as the organic linker in the hybrids. The gel extracted DNA–TPA conjugates were used for further downstream studies including self-assembly. A similar procedure was followed for the coupling of S2 ssDNA with TPA. PAGE analysis indicates a similar analysis profile (see Supporting Information File 1, Figure S1).

The covalent conjugation of 2,6,14-TPA with amine-modified DNA was confirmed by RP-HPLC and MALDI-TOF analysis. In RP-HPLC profile, the first two peaks were found to be very close to each other (see Supporting Information File 1, Figure S2 and Figure S3). The first peak having the lowest retention time corresponds to unreacted DNA. This is followed by the mono-, di- and triconjugated DNA–TPA hybrids. The insignificant difference in the retention time for S1–TPA conjugates as compared to S2–TPA is due to the differences in the nucleotide content of S1 and S2.

MALDI-TOF MS was performed with PAGE-purified DNA–TPA hybrid conjugates. The measured mass values for the S1–TPA conjugates at 8301 Da (calculated mass value = 8301.5 Da) and 12200 Da (calculated mass value = 12223 Da) correspond to the di-conjugated (S1)_2_–TPA and triconjugated (S1)_3_–TPA, respectively (see Supporting Information File 1, Figure S4 and Figure S5). Similarly, MALDI-TOF peaks for S2–TPA conjugates were found at 8426 Da (calculated mass value: 8426 Da) for the disubstituted conjugates and 12398 Da (calculated mass value: 12409 Da) for the trisubstituted conjugates, respectively (see Supporting Information File 1, Figure S6 and Figure S7). Thus, the formation of both S1 and S2 DNA–TPA covalent di- and triconjugates were unambiguously established by using RP-HPLC and MALDI-TOF.

### Self-assembly of DNA–TPA hybrid molecular building blocks

**Figure 2:**
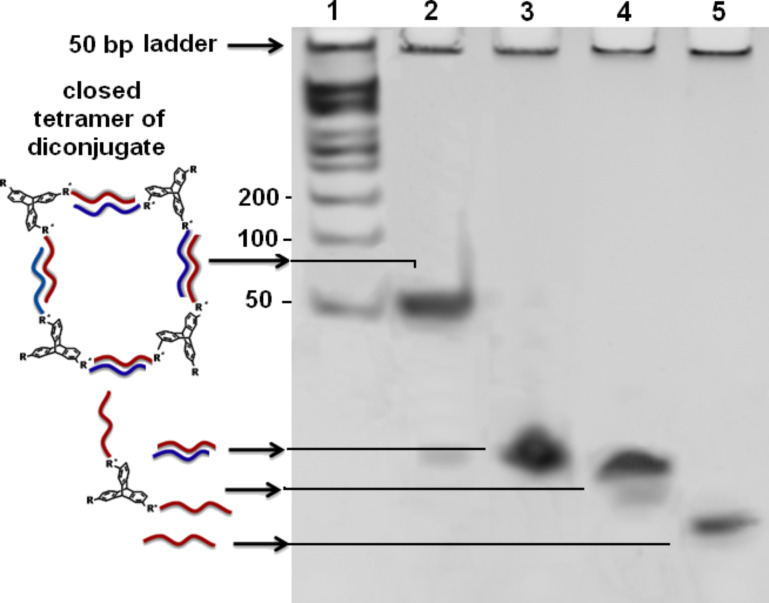
Native PAGE image (12%) of self-assembly of dicojugate DNA–TPA units with 2 μM total ssDNA concentration. Lane 2 shows the assembly of DNA–TPA diconjugates from (S1)_2_–TPA and (S2)_2_–TPA.

The DNA–TPA diconjugates (S1)_2_–TPA and (S2)_2_–TPA were self-assembled by hybridization in the presence of a buffer. The self-assembled products were observed with native PAGE and compared with molecular markers and the parent hybrid S1–S2. It was expected that self-assembly of (S1)_2_–TPA and (S2)_2_–TPA would give rise to a linear 1D array, where the complementary hybrid building blocks would be alternatively placed. This would produce higher-ordered smeared bands resulting from a wide distribution of the linear array. However, such products were not found with native PAGE. The self-assembly of the diconjugated hybrids lead to the formation of a single product as evident from the appearance of a single band in the native PAGE (Figure 2). This product corresponds to the self-assembled closed tetrameric unit in which two units of each (S1)_2_–TPA and (S2)_2_–TPA are involved.

Apart from native PAGE, the formation of these closed structured products was further confirmed by the treatment of the product with the enzyme mung bean nuclease (MBN). This enzyme is very selective for ssDNA where the digestion of dsDNA is negligible at approximately 30,000:1 (ssDNA:dsDNA). The fact that band mobility remains unchanged before and after MBN treatment ratifies the absence of any unhybridized ssDNA (see Supporting Information File 1, Figure S8) and further supports the formation of a closed and confined structure. The hybridization in the presence of Zn PpIX does not have any notable effect on the tetrameric structures. This is evident from the similar mobility of the corresponding band in PAGE where Zn PpIX has been added to the diconjugated self-assembly. Thus, closed tetrameric products are the most stable products of diconjugate building blocks that restrict further self-assembly to give a continuous nanostructure of higher dimensions.

**Figure 3:**
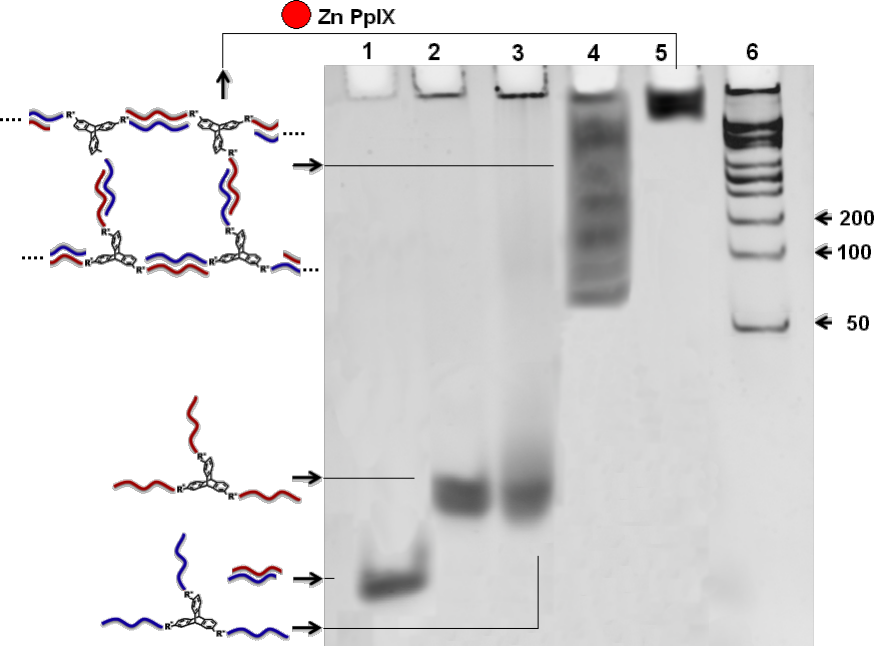
Native PAGE-gel image (8%) of self-assembled triconjugated DNA–TPA units with 2 μM total ssDNA concentration. Lane 5 shows the self-assembly of triconjugates in the presence of Zn PpIX.

Hybridization-mediated self-assembly of the DNA–TPA triconjugates (S1)_3_–TPA and (S2)_3_–TPA leads to the formation of ordered structures in the presence and absence of Zn PpIX. These higher-ordered structures were evident in native PAGE where few dominant bands were observed in the absence of Zn PpIX, which are corresponding to self-assembled DNA structures in the range of 100–1000 base pairs with respect to the molecular markers. Interestingly, there is a drastic change in the product distribution when the hybridization takes place in the presence of Zn PpIX. These self-assembled DNA structures hardly move in the gel and were found to accumulate beneath the loading wells of the native PAGE. This suggests that Zn PpIX influences the self-assembly to re-equilibriate the product formation towards the development of more organized DNA structures with large dimensions (Figure 3). However, it was observed that the mere addition of Zn PpIX to the DNA–triptycene conjugates at room temperature does not give rise to higher-ordered structures even after 24 h of co-incubation. Interestingly, when the triconjugates are heated to 90 °C in the presence of Zn PpIX and immediately cooled on dry ice, discrete structures are formed by this method and ordered structures are scanty (see Supporting Information File 1, Figure S9). MBN treatment has no effect on the self-assembly in the presence or absence of Zn PpIX, thereby ruling out the possibility of smaller units tagged with unhybridized ssDNA tails (see Supporting Information File 1, Figure S10).

### Dynamic light scattering studies of DNA–TPA triconjugate self-assembly

The average size distributions of the self-assembled DNA–TPA di- and triconjugates in the presence and absence of Zn PpIX were evaluated by DLS (Table 2 and Supporting Information File 1, Figure S11). Similar to observations of native PAGE, a narrow size distribution was observed in DLS indicating the formation of a single product for the self-assembled diconjugates. The size distribution remains virtually unchanged, when the hybridzation takes place in the presence of Zn PpIX. The size distribution after the self-assembly of DNA–TPA triconjugates is broad with a high polydispersity index (PDI) whereas the PDI decreases in the presence of Zn PpIX. A narrow size distribution for the coassembly of DNA–TPA triconjugates with Zn PpIX correlates well with the appearance of higher-ordered structures in the native PAGE.

**Table 2:** Hydrodynamic size (nm) and PDI values for different systems obtained from number distribution analysis of DLS data. Zn PpIX was added during assembly.

self-assembled system	average hydrodynamic radius (nm)	PDI

Zn PpIX	2.6 ± 0.7	0.75
S1 S2 DNA duplex + Zn PpIX	96 ± 25	0.66
DNA–TPA diconjugates	169 ± 39	0.28
DNA–TPA diconjugates + Zn PpIX	178 ± 27	0.25
DNA–TPA triconjugates	900 ± 105	0.8
DNA–TPA triconjugates + Zn PpIX	1500 ± 78	0.23

### AFM imaging and analysis of self-assembled DNA nanostructures

**Figure 4:**
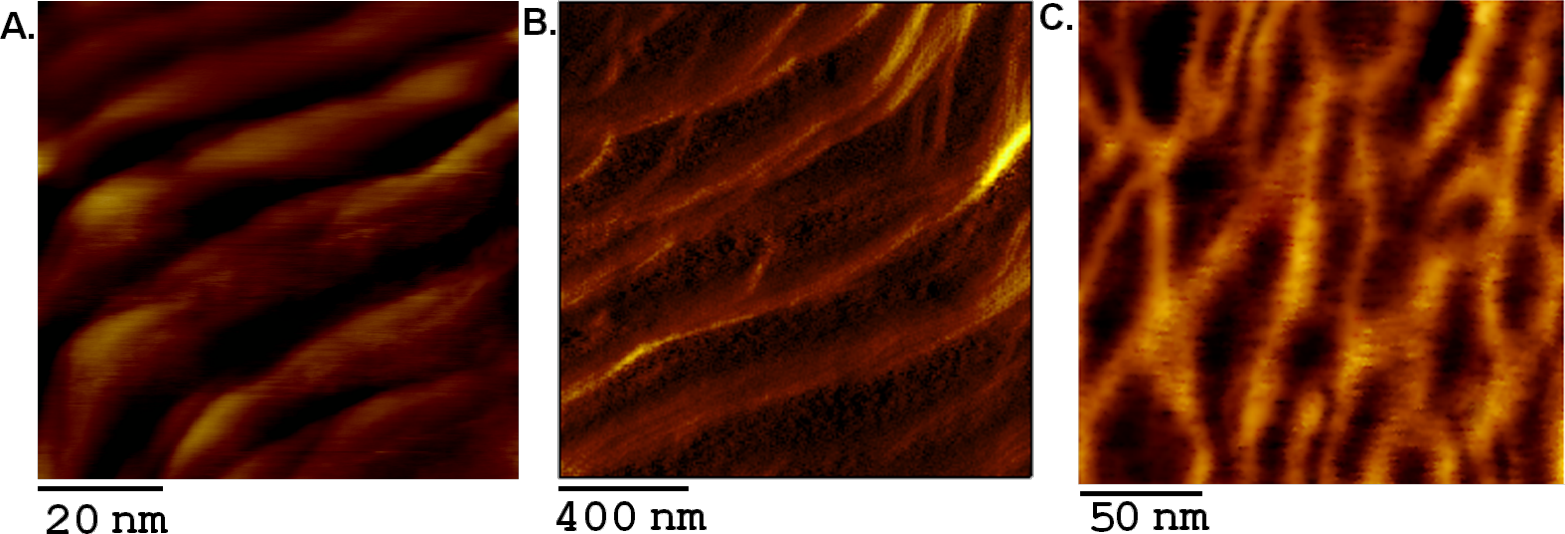
AFM images of the self-assembly of DNA–TPA tri-conjugates. A and B in the presence of Zn PpIX and C in the absence of Zn PpIX.

AFM imaging was employed for the direct visualization of the self-assembled (S1)_3_–TPA and (S2)_3_–TPA hybrid conjugates. AFM images show the formation of extended nanofibers through the self-assembly into hierarchically organized structures of DNA–TPA units, when hybridized in the presence of Zn PpIX. The presence of Zn PpIX helps in the alignment of the nanofibers (Figure 4 and Supporting Information File 1, Figure S13).The nanofibers have a width of 9–15 nm, which correlates well with calculations from modeling methods.The interaction of Zn PpIX with DNA by outside stacking along the helix significantly increases the stability of the DNA duplex and simultaneously provides a template to initiate the formation of nanofibers [50]. The nanofibers appear to be compactly organized and bundled together in the form of elongated rope-like structures. However, in the absence of Zn PpIX during the self-assembly process leads to the formation of an ill network (Supporting Information File 1, Figure S12). This is attributed to hybridization defects that disturb the periodicity.

### Modeling studies

**Figure 5:**
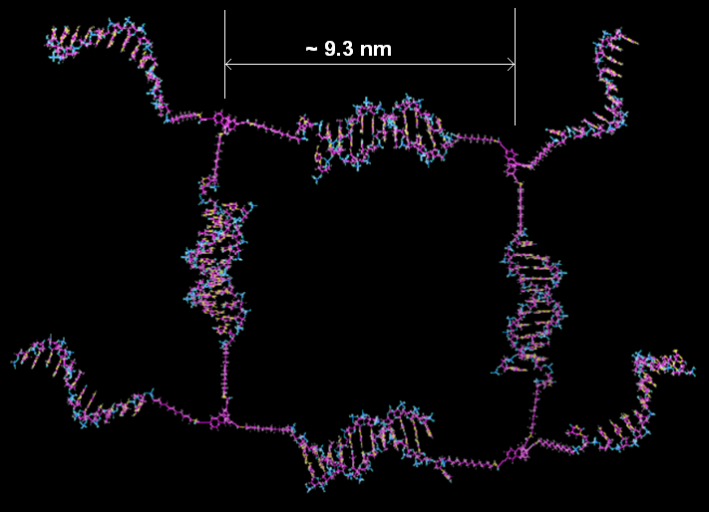
Modeling studies involving S1–TPA and S2–TPA triconjugates showing a single tetrameric unit with square structure having extended ssDNA arms at the corner. The vertex length of the structure was found to be ca. 9.3 nm.

The newly assembled DNA–TPA nanostructures were further formulated through computational analysis where the precise dimension of the basic units of the nanofiber/nanoladder is proposed. We sketched the structure of tetramers of S1/S2 DNA–TPA di- and triconjugates (Figure 5, see Supporting Information File 1, Figure S14–S16). The formation of the tetramer results from the hybridization of four DNA–TPA triconjugates (two units each of S1–TPA and S2–TPA triconjugates). Two out of three ssDNA strands of each of the DNA–TPA triconjugates participate in the formation of a single tetrameric unit. One ssDNA arm at each corner of the closed tetramer is available for hybridization with another DNA–TPA triconjugate. The in silico model represents the conjugation of 2,6,14-triptycenetripropiolic acid (TPA) with ssDNA to form the fiber-like composite DNA nanostructure. This model explains how each repeating unit of TPA coupled with ssDNA would associate through H-bonding between the bases to form DNA nanofibers. The structure was preprocessed, wherein appropriate bond angles were assigned and missing hydrogens were added. Although the dynamics of the structure over a fixed period of time was not studied, we have optimized and assigned proper geometry to the structure using OPLS_2005 force field (Maestro9.9, Schrödinger, LLC, New York, NY, 2014). Thus, using an in silico modeling approach, we have generated a three-dimensional structure of TPA conjugated DNA, which was further used to calculate the parameters such as surface area and volume of the structure.

The total surface area and the volume of this tetrameric unit (single ladder step) were found to be ca. 130.5 nm^2^ and ca. 348 nm^3^, respectively, calculated by using the tool 3V: cavity, channel and cleft volume calculator and extractor [51]. The surface area of a single Zn PpIX molecule has been also calculated (8.35 nm^2^). The calculations provide a hint about the maximum number of Zn PpIX molecules that could be accommodated in a single step of the ladder (tetrameric unit), which is about 15 without considering any non-covalent interaction between Zn PpIX itself. This study shows the formation of complex structures proceeds through a step-wise self-assembly of tetrameric triconjugates and the subsequent periodic growth when hybridized in the presence of Zn PpIX.

### Thermal melting analysis

**Figure 6:**
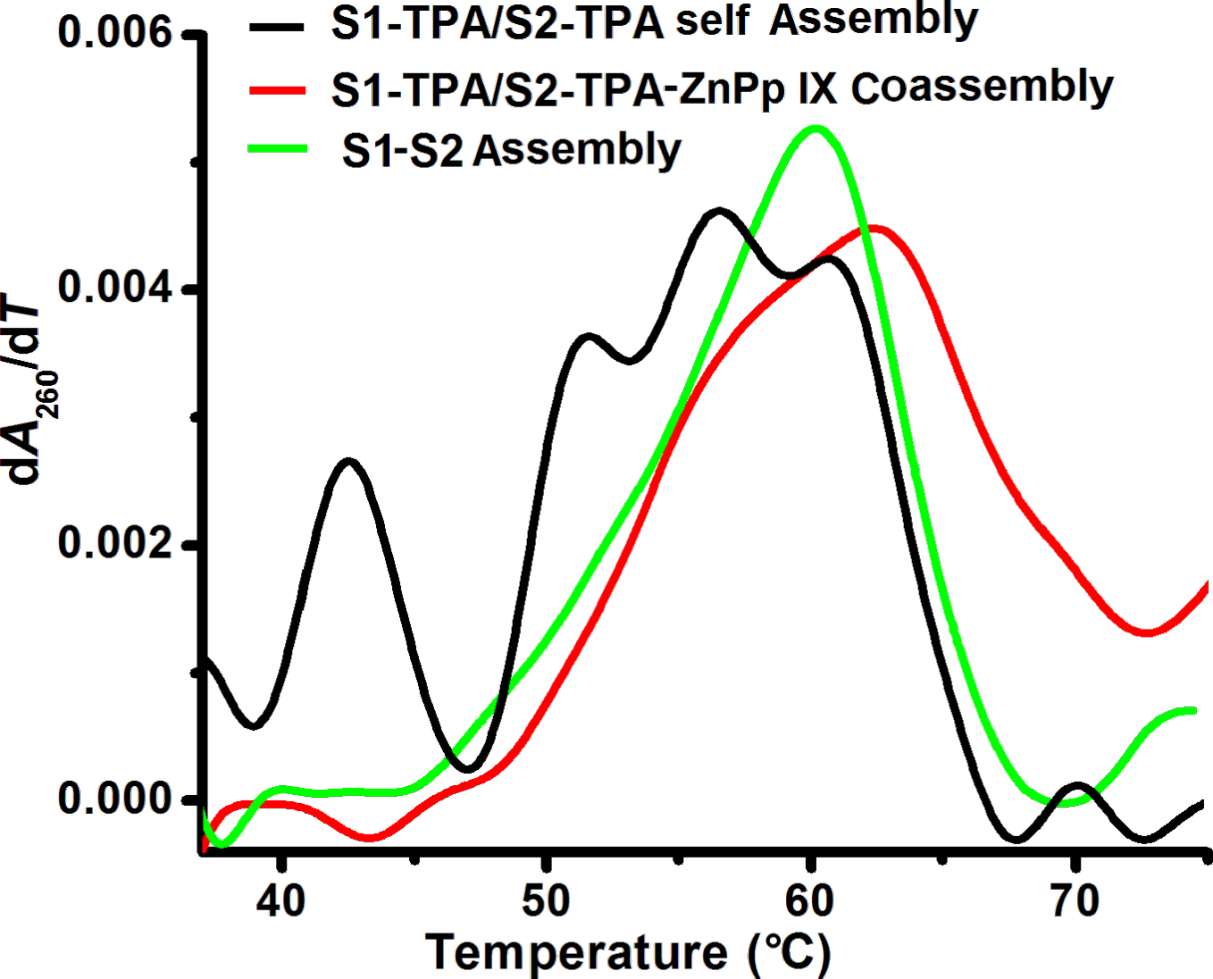
The first-order-derivative melting curves of nanofibers (S1 DNA–TPA/S2 DNA–TPA triconjugate Zn PpIX coassembly) from self-assembly of DNA–TPA triconjugates from absorbance of DNA in solution at 260 nm.

The formation of stable higher-ordered DNA nanostructures through the coassembly of DNA–TPA triconjugates with Zn PpIX was also evaluated by temperature-controlled UV absorption measurements. This analytical technique is a valuable tool for obtaining a better understanding of the assembly of complex DNA nanostructures from short oligonucleotide–organic hybrid molecule conjugates, which associate with each other according to an assembly plan encoded in their sequences. Reportedly, the thermal melting of DNA nanostructures depends upon several parameters such as concentration of DNA, the hierarchy of the assembly, annealing protocol and distance between two parallel strands of DNA [5,52]. Under our experimental conditions, the melting transition was observed between 50 and 65 °C for the S1–S2 duplex, it is between 30 to 65 °C, between 40 and 65 °C for the assembly of TPA–DNA triconjugates in the absence and in the presence of Zn PpIX, respectively. The melting temperature, *T*_m_, of S1–S2 is about 59 °C and about 65 °C for the coassembly of DNA–TPA triconjugates and Zn PpIX (Figure 6). The increase in *T*_m_ indicates the formation of a self-assembled ordered structure where DNA duplexes are closely packed and highly oriented. The increase in *T*_m_ is also ascribed to the combination of stacking of Zn PpIX along the DNA duplex, reduced configurational entropy and ion-cloud sharing [8,53]. However, the first-order derivative of the self-assembled DNA–TPA triconjugates is broad in the absence of Zn PpIX, with multiple melting transitions extending over the entire temperature range. This result clearly indicates the formation of ill-formed networks of DNA–TPA triconjugates that transform into more orderly structures after the addition of Zn PpIX.

### Chiroptical properties of nanofibers

**Figure 7:**
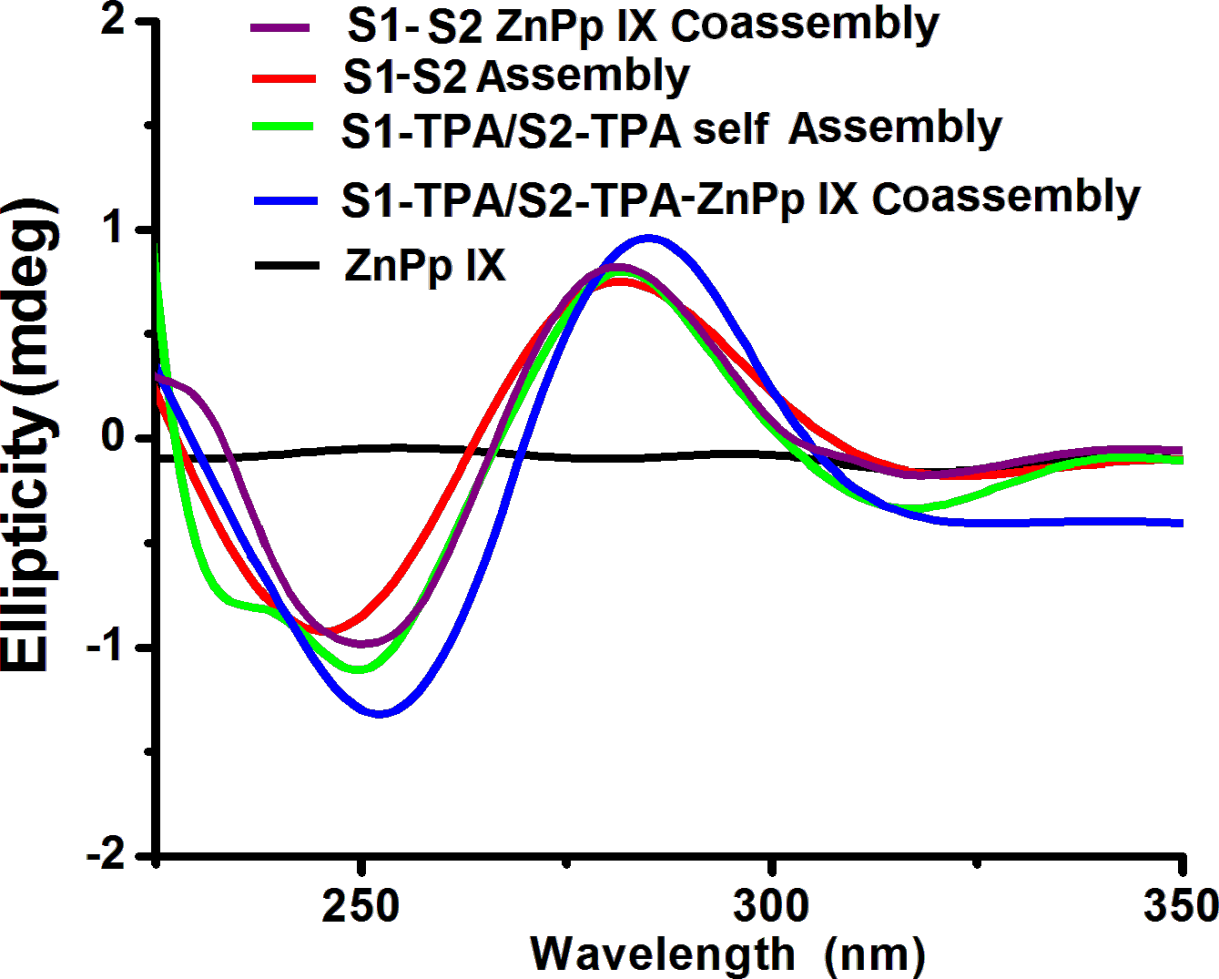
CD spectra showing the chirality and conformation of nanofiber (S1 DNA–TPA/S2 DNA–TPA triconjugate Zn PpIX coassembly) and their controls.

Chiral nanostructures have raised significant interest among materials scientists, because of their application in chiral memory, data storage, biological sensing and optical communication technology [54]. Along with these, the primary inspiration for the development of chiral nanomaterials is the opportunity to create chiral metamaterials with negative refractive indices [55]. Reportedly, chiral nanostructures are constructed using chiral templates where DNA is frequently employed [56]. Hence, the positioning of Zn PpIX in a DNA–TPA scaffold stipulates the study of induced chirality in the above constructed nanostructure. The chirality and conformational changes induced in DNA after conjugation with TPA as well as after the self-assembly and coassembly with Zn PpIX was evaluated by CD analysis. Distinct CD signals were observed in the UV–visible part of the spectrum from 200 to 650 nm for dsDNA and the nanofiber, whereas negligible CD signals were observed for free Zn PpIX (Figure 7). This can be attributed to the formation of chiral DNA nanostructures. The induced CD signal from 400–600 nm is attributed to the outside stacking of Zn PpIX molecules along the helix of the DNA–TPA self-assembly structure that is expected for this system (see Supporting Information File 1, Figure S17). A strong bisignate CD signal in the ultraviolet part of the spectrum in the range of 200–280 nm clearly show that DNA duplex maintained a B-conformation and thus Watson–Crick base pairing is sustained in these self assembled chiral nanoscale superstructure. However, change in peak intensity and shape of the CD spectra for different structures is due to the possible changes in the average turn per base of DNA and the constituents of the system resulting from conformational strain.

### Catalytic activity of composite DNA nanostructures

**Figure 8:**
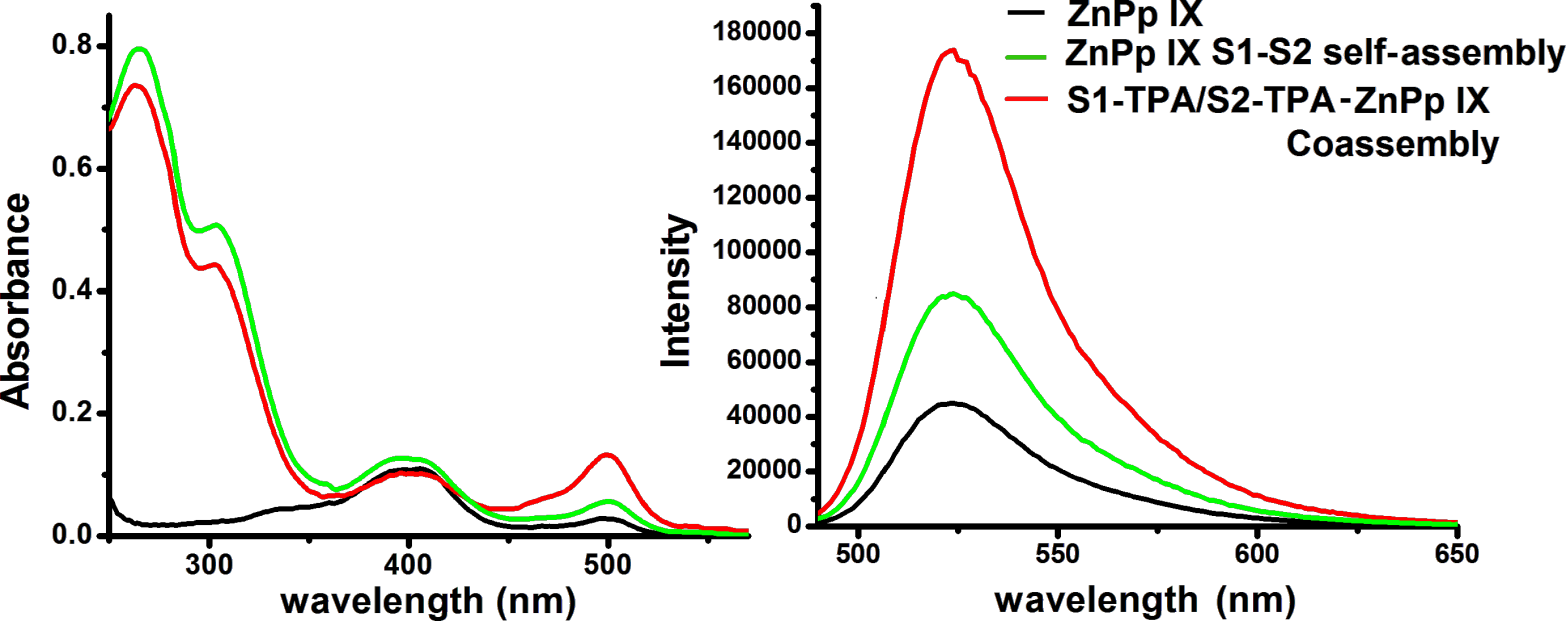
UV–vis absorption spectra and steady-state fluorescent spectra of rhodamine 123 quantifying the photocatalytic activity of nanofibers (S1 DNA–TPA/S2 DNA–TPA triconjugate Zn PpIX coassembly) and their control.

Considerable research efforts in the direction of controlled and improved ROS generation are being conducted for application in photodynamic therapy (PDT), decontamination of water and others [57–58]. We have constructed a composite DNA–TPA-based hybrid nanostructure, which displays enhanced ROS generation and at the same time is biocompatible. To ascertain the elevated generation of ROS, the oxidation of DHR 123 into rhodamine 123 (R 123) in the presence of ROS was chosen as a prototype where ROS generation is proportional to the extent of oxidation of DHR 123. The ROS generation was attributed to energy transfer from the PpIX molecules to neighboring oxygen atoms upon irradiation at 330 nm. The formation of R 123 from DHR 123 was quantified by steady-state fluorescence (λ_em_ = 530 nm) and absorption spectra (λ_abs_ = 500 nm). We found that at a given concentration of DHR 123 and a fixed irradiation time, the composite DNA nanofibers that were constructed from assemblies of DNA–TPA triconjugates in the presence of Zn PpIX produced the maximum ROS as compared to a simple DNA–Zn PpIX mixture or free Zn PpIX in solution (Figure 8). Over the course of the entire reaction time, the enhancement in the formation of R 123 by the composite DNA nanofiber (DNA–TPA triconjugate–Zn PpIX coassembly) and DNA–Zn PpIX is ca. 79% and 45% respectively compared to free Zn PpIX. The main aim of the experiment is to show that at a given concentration of Zn PpIX and DNA, the ROS generation is higher in the organized system of S1 DNA–TPA/S2 DNA–TPA triconjugate Zn PpIX coassembly. Hence, the presence of internal ordering of the nanostructure does indeed influence the catalytic activity. These results are encouraging for PDT and other applications, where organized DNA structures can be considered for dose-dependent delivery of ROS in relevant systems.

## Conclusion

A novel DNA–organic hybrid molecule has been synthesized by the covalent coupling of amine-terminated DNA with TPA. Characterization of the DNA–TPA hybrids by denaturing PAGE, RP-HPLC and MALDI-TOF analysis showed the formation of all three possible products in which TPA was conjugated with either one, two or three ssDNA. The rigid framework of TPA is expected to produce scaffolds for the biologically relevant molecule (Zn PpIX) after conjugation and assembly with DNA complementary strands. Interestingly, coassembly of DNA–TPA building block units and Zn PpIX generates DNA nanofibers showing enhanced photocatalytic activity. These features have been identified and confirmed by native PAGE, AFM, CD and spectroscopic analyses. It was observed that tri-conjugated hybrid units are self-assembled into small oligomeric products leading to unorganized structures in the absence of Zn PpIX where as higher-order organized structures with B-form conformation of DNA were seen in the presence of Zn PpIX. Although, the TPA moiety offers 120° angular disposition of the ssDNA strands after conjugation, tetrameric building blocks are still formed due to the inherent flexibility of the DNA duplex after self-assembly. It can be concluded that the Zn PpIX re-equilibrate the self-assembled mixture into the selected nanostructures, thus providing an additional level of control in DNA structuring. Our experiments point out to the fact that Zn PpIX redirects the self-assembly and initiates the formation of ordered structures. In fact, in the absence of Zn PpIX during the annealing process, triconjugates are restricted to ill-formed network structures. Furthermore, the long-range alignment of Zn PpIX in preorganised systems has enhanced the oxidation of the ROS scavenger DHR 123 as compared to free Zn PpIX. Therefore, this type of nanostructure provides unprecedented opportunities to design uniform and safe PDT devices with precise structures, tailorability, high efficacy and biological relevancy. Conjugation of TPA with oligomeric DNA results in tuned material property and porosity of the nanostructures. Such methodology offers a new opportunity for the construction of composite nanostructures by the positioning of a guest molecule on DNA–TPA hybrid molecule scaffolds. The structural feature of DNA such as cavities and clefts, and the internal free volume of triptycene molecules may have a significant influence on the positioning and the functional properties of the guest molecules in these composite nanostructures.

## Supporting Information

Supporting information contains characterization of DNA–TPA conjugates and assemblies in presence and absence of Zn PpIX. This file contains denaturing and native PAGE, RP-HPLC chromatograms, MALDI mass spectrometry spectra, AFM images, CD spectra and some computational data.

File 1 Characterization of DNA–TPA conjugates.
