# Supplementary material for: Hierarchical coassembly of DNA–triptycene hybrid molecular building blocks and zinc protoporphyrin IX
Source: Beilstein J Nanotechnol. 2016 May 12;7:697–707. doi: 10.3762/bjnano.7.62 (PMC4901925; doi:10.3762/bjnano.7.62)
Supplement: File 1 — Characterization of DNA–TPA conjugates. [file Beilstein_J_Nanotechnol-07-697-s001.pdf]

Supporting Information

for

Hierarchical coassembly of DNA–triptycene hybrid molecular building blocks and zinc protoporphyrin IX

Rina Kumari[1], Sumit Singh[1], Mohan Monisha[2], Sourav Bhowmick[1], Anindya Roy[2], Neeladri Das*,§1 and Prolay Das*,§1

Address: [1]Department of Chemistry, IIT Patna, Bihata 801118, India and [2]Department of Biotechnology, IIT Hyderabad, Hyderabad,502205, India

Email: Prolay Das* - prolay@iitp.ac.in; Neeladri Das* - neeladri@iitp.ac.in

* Corresponding author

§Tel: +91 612 255 2057, +91 9631624108; Fax: +91 612 257 7383

1. Characterization of DNA–TPA conjugates

Synthesis of succinimidyl-activated 2,6,14-triptycenetripropiolate ester

TPA is activated through DCC/NHS-mediated cross coupling route in which 110 µmol of 2,6,14-TPA with 330 µmol DCC and 495 µmol NHS were mixed in 1 mL DMF and stirred for 12 h at room temperature. A pellet of dicyclohexyl urea (DCU) was separated by centrifugation and the supernatant containing the active ester was used for further reaction.

S2 DNA–TPA hybrid conjugates:

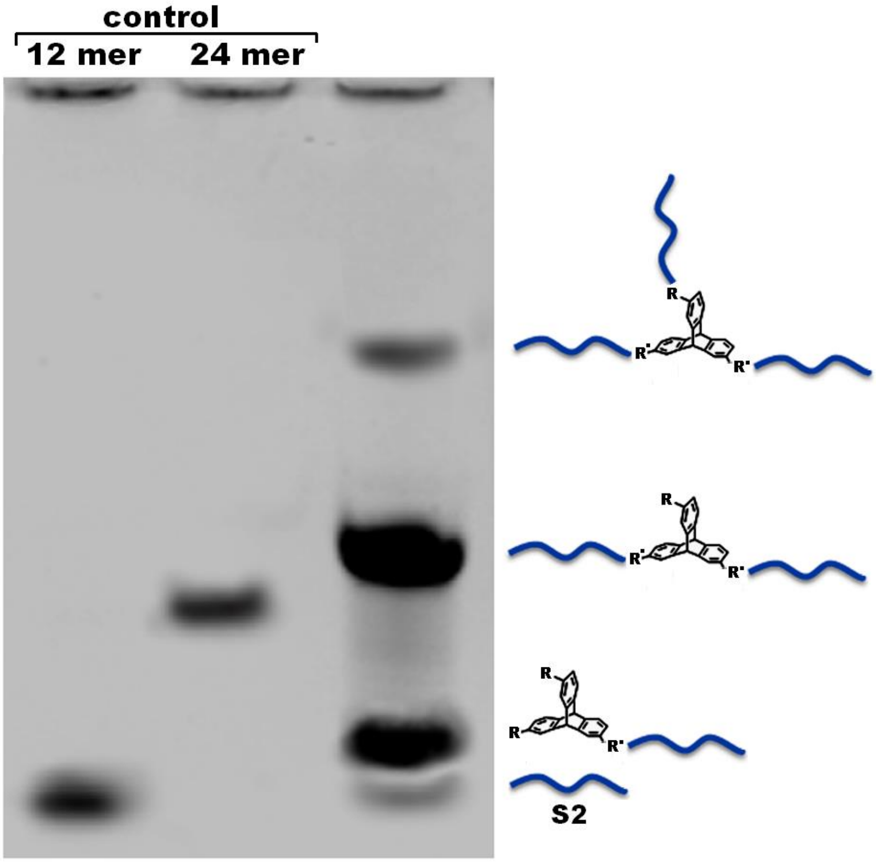

Figure S1: 20% denaturing PAGE analysis of DNA (S2)–TPA conjugates showing decrease in gel mobility of the conjugates upon successive conjugation of ssDNA strands to the triptycene core.

HPLC and MALDI-TOF analysis of ssDNA–triptycenetripropiolate conjugates:

The conjugation of amine DNA with TPA was also confirmed by RP-HPLC and MALDI-TOF analysis. The reverse-phase HPLC (Shimadzu-VP, Kyoto, Japan) having a spinco tech-C18Q column (50 × 4.60 mm) was used for analysis. Linear gradient elution was used with 5–100% acetonitrile (CH_3CN) in 100 mM triethylammonium acetate (TEAA) buffer (pH 7.4) over 60 min at a flow rate of 1 mL/min with UV detection at 260nm. The reaction mixture was diluted in 0.1 M TEAA buffer and 20 µL of the sample was injected in the injector. The the dialyzed reaction mixture was subjected to molecular weight determination by Autoflex II MALDI-TOF-MS (Bruker Daltonics, Billerica, MA) using the linear positive mode. Data processing was performed by Flex Analysis software.

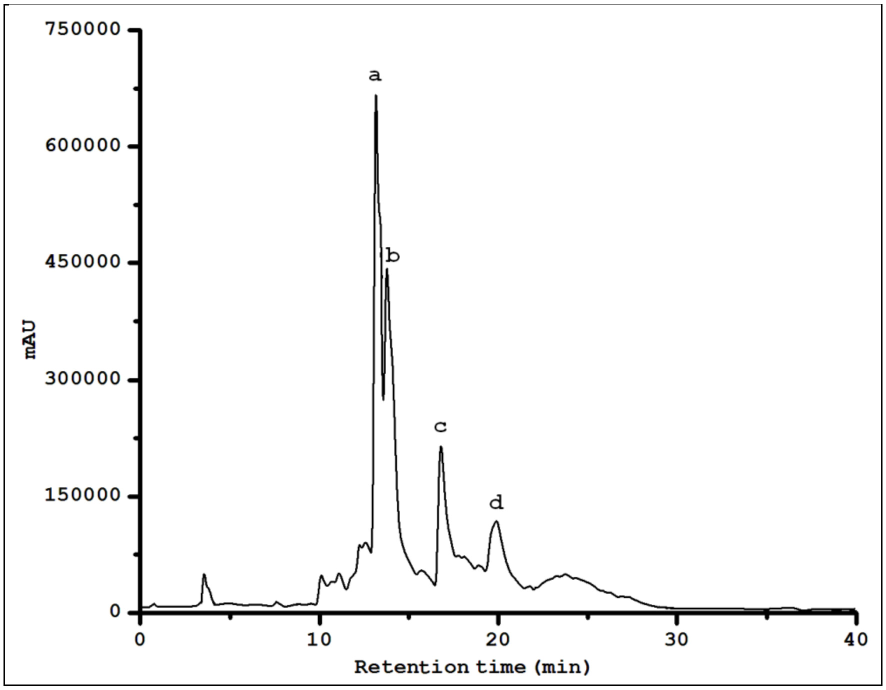

Figure S2: Analytical RP-HPLC profiles of S1 DNA–TPA reaction mixture; a, b, c and d correspond tothe unreacted, monoconjugate, diconjugate and triconjugate, respectively.

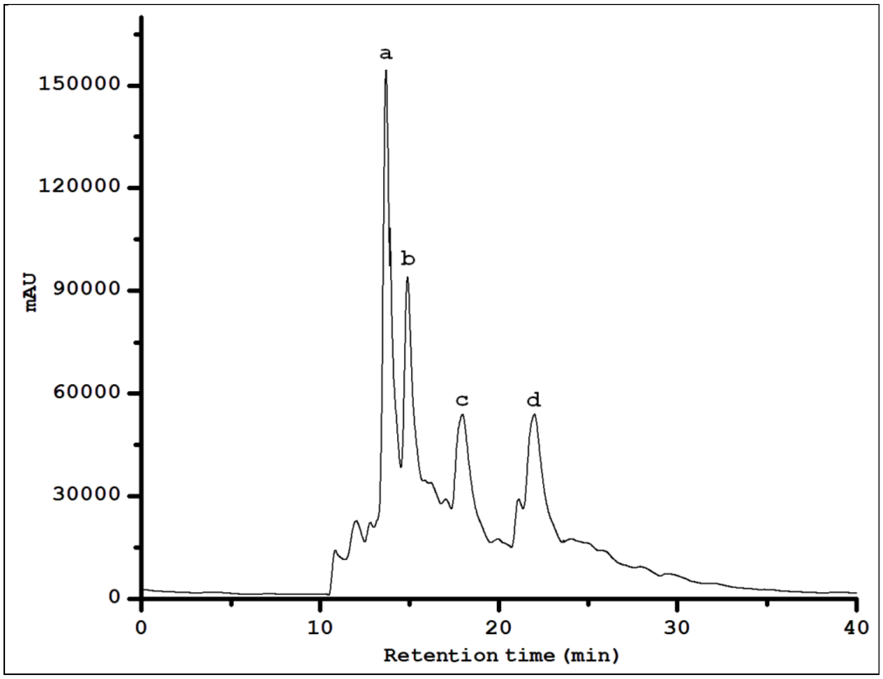

Figure S3: Analytical RP-HPLC profiles of S2 DNA–TPA reaction mixture; a, b, c and d correspond unreacted, monoconjugate, diconjugate and triconjugate, respectively.

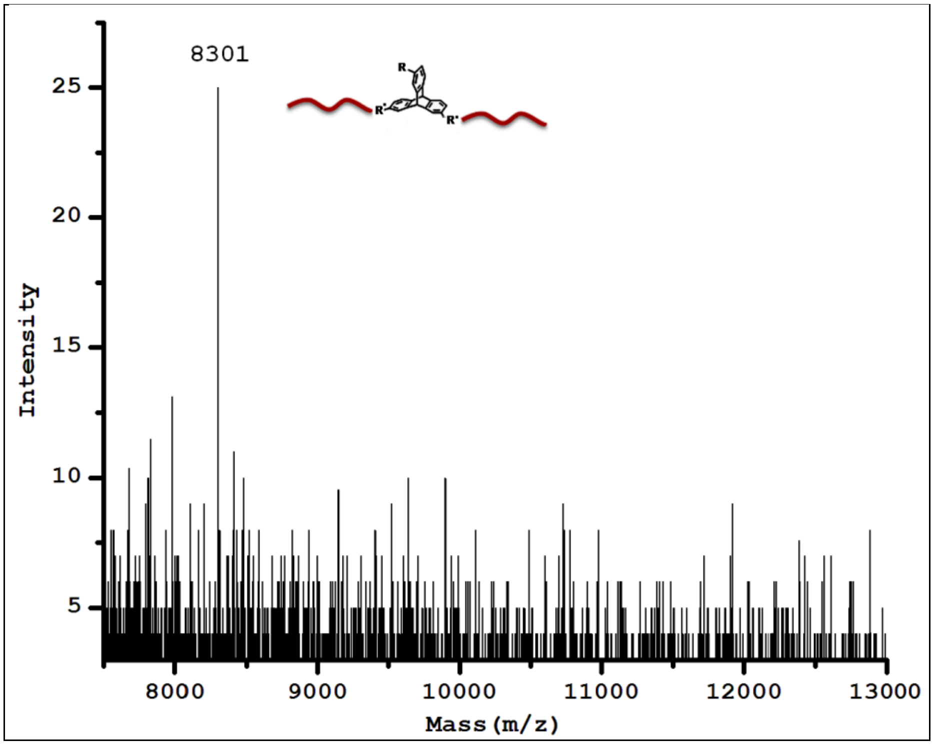

Figure S4: MALDI-TOF spectra of S1 DNA–TPA diconjugates ((S1-DNA)$_2$–TPA) after extraction and purification with PAGE, the observed mass of (S1-DNA)$_2$–TPA is m/z = 8301 and the calculated mass is m/z = 8301.5.

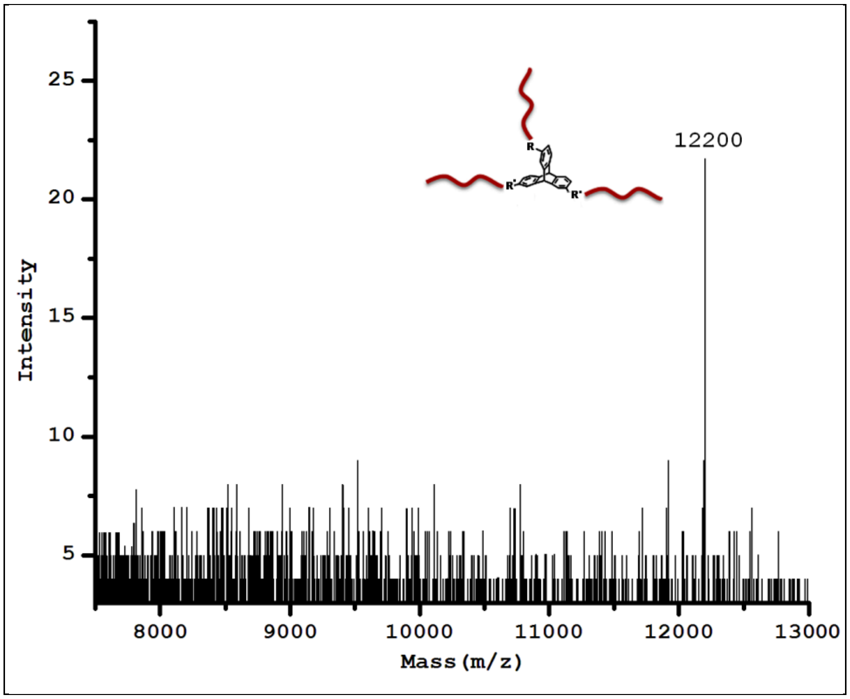

Figure S5: MALDI-TOF spectra of S1 DNA–TPA triconjugates ((S1-DNA)$_3$–TPA) after extraction and purification with PAGE, the observed mass of (S1-DNA)$_3$–TPA is *m*/*z* =12200 and the calculated mass is *m*/*z* =12223.

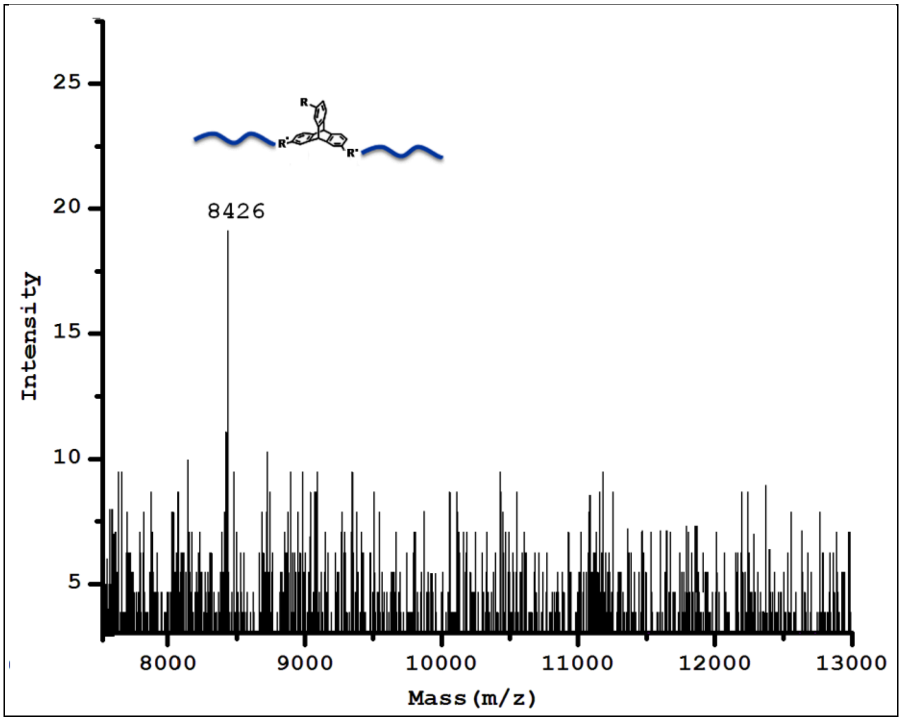

Figure S6: MALDI-TOF spectra of S2 DNA–TPA diconjugates ((S2-DNA)$_2$–TPA) after extraction and purification with PAGE, the observed mass of (S2-DNA)$_2$–TPA is *m*/*z* = 8426 and the calculated mass is *m*/*z* = 8426.

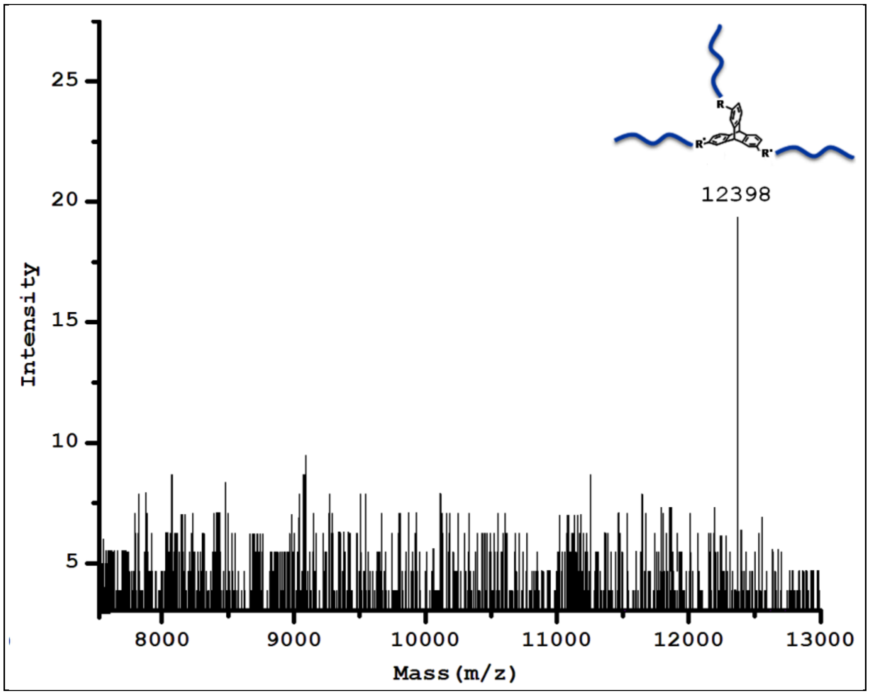

Figure S7: MALDI-TOF spectra of S2 DNA–TPA triconjugates ($(S2\text{-}DNA)_3$–TPA) after extraction and purification with PAGE, the observed mass of $(S2\text{-}DNA)_3$-TPA is m/z = 12398 and the calculated mass is m/z = 12409.

2. Characterization of DNA-TPA self assemblies

Mung bean nuclease (MBN) digestion

The cyclic and double stranded form of DNA in nanostructures has been resolved by using mung bean nuclease (MBN) digestion assays. Under optimized conditions, MBN degrades linear open structures that contain ssDNA, but does not degrade dsDNA. The ratio of degradation of ssDNA over dsDNA are 30,000:1. After the hybridization of diconjugated ($(S1)_2$–TPA and $(S2)_2$–TPA) and triconjugated ($(S1)_3$–TPA and $(S2)_3$–TPA)) building blocks, MBN digestion was performed with 20 units of the enzyme in MBN buffer of pH 5.0 at 37 °C for 30 min prior to introducing them into PAGE.

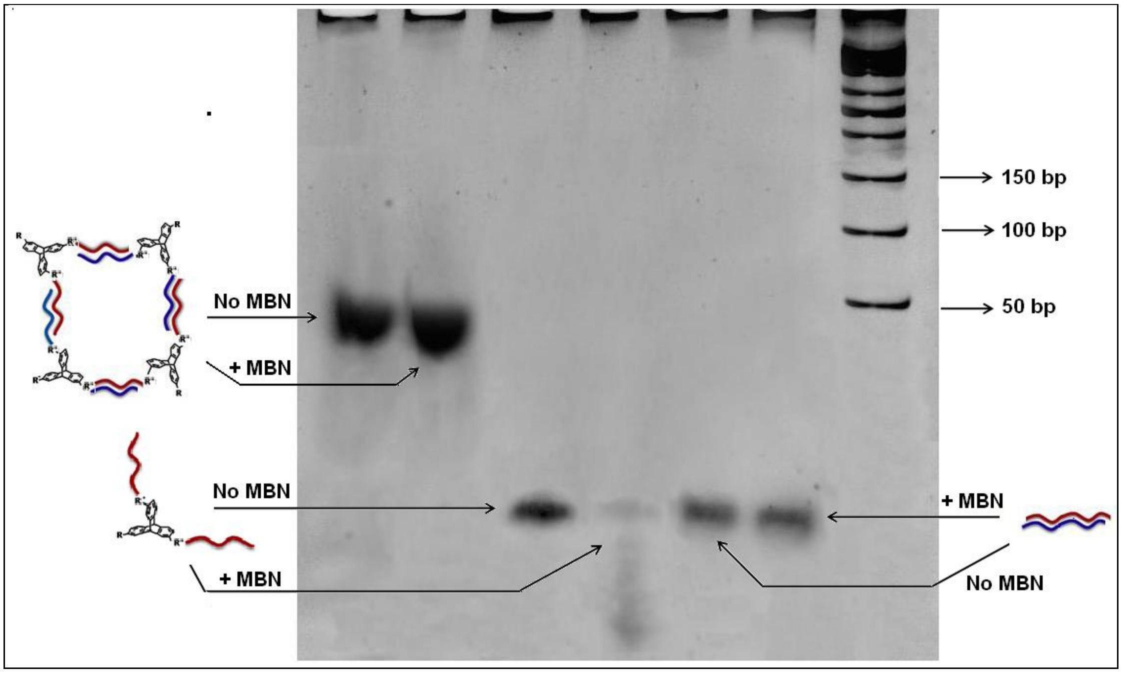

Figure S8: 10% Non-denaturing PAGE to show MBN digestion assay for the assemblies generated from diconjugated DNA–TPA building blocks.

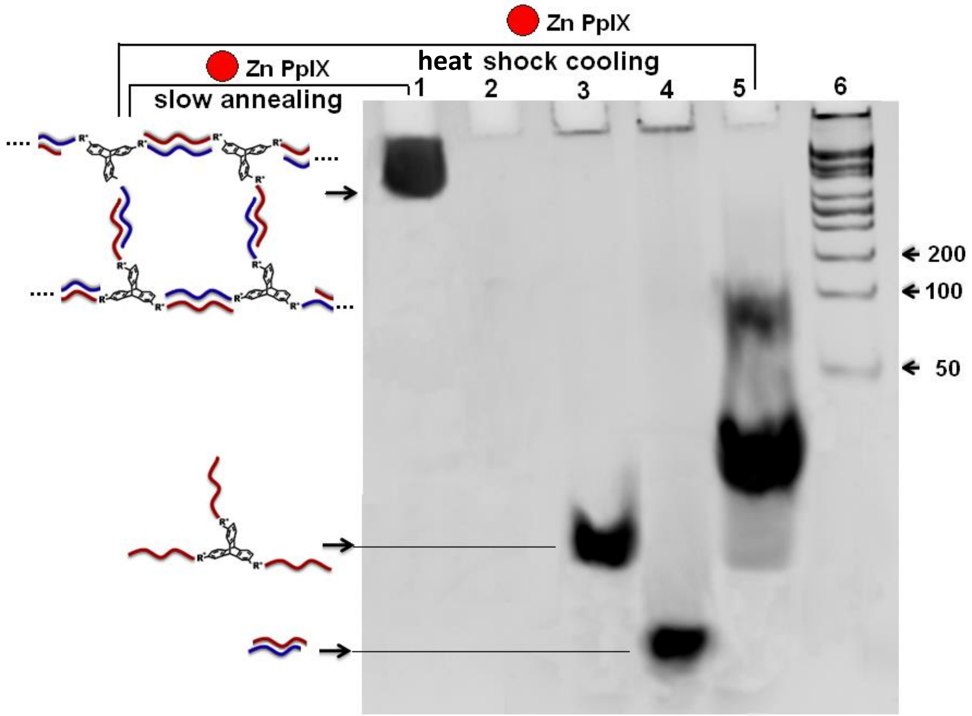

Figure S9: Native PAGE image (8%) of self- assembled triconjugated DNA–TPA units showing difference in gel mobility due to slow annealing and heat shock cooling in the presence of Zn PpIX.

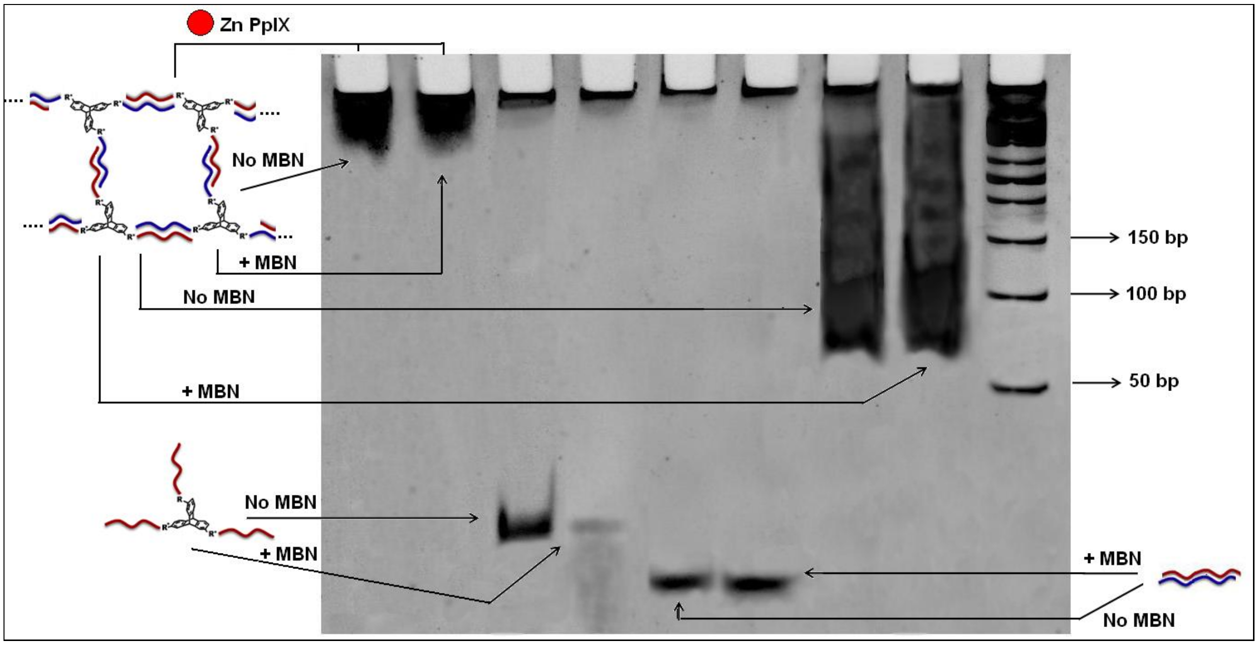

Figure S10: 8% Non-denaturing PAGE to show products of MBN digestion of the assemblies of triconjugated DNA–TPA building blocks in the absence and presence of Zn PpIX.

Dynamic light scattering (DLS) analysis:

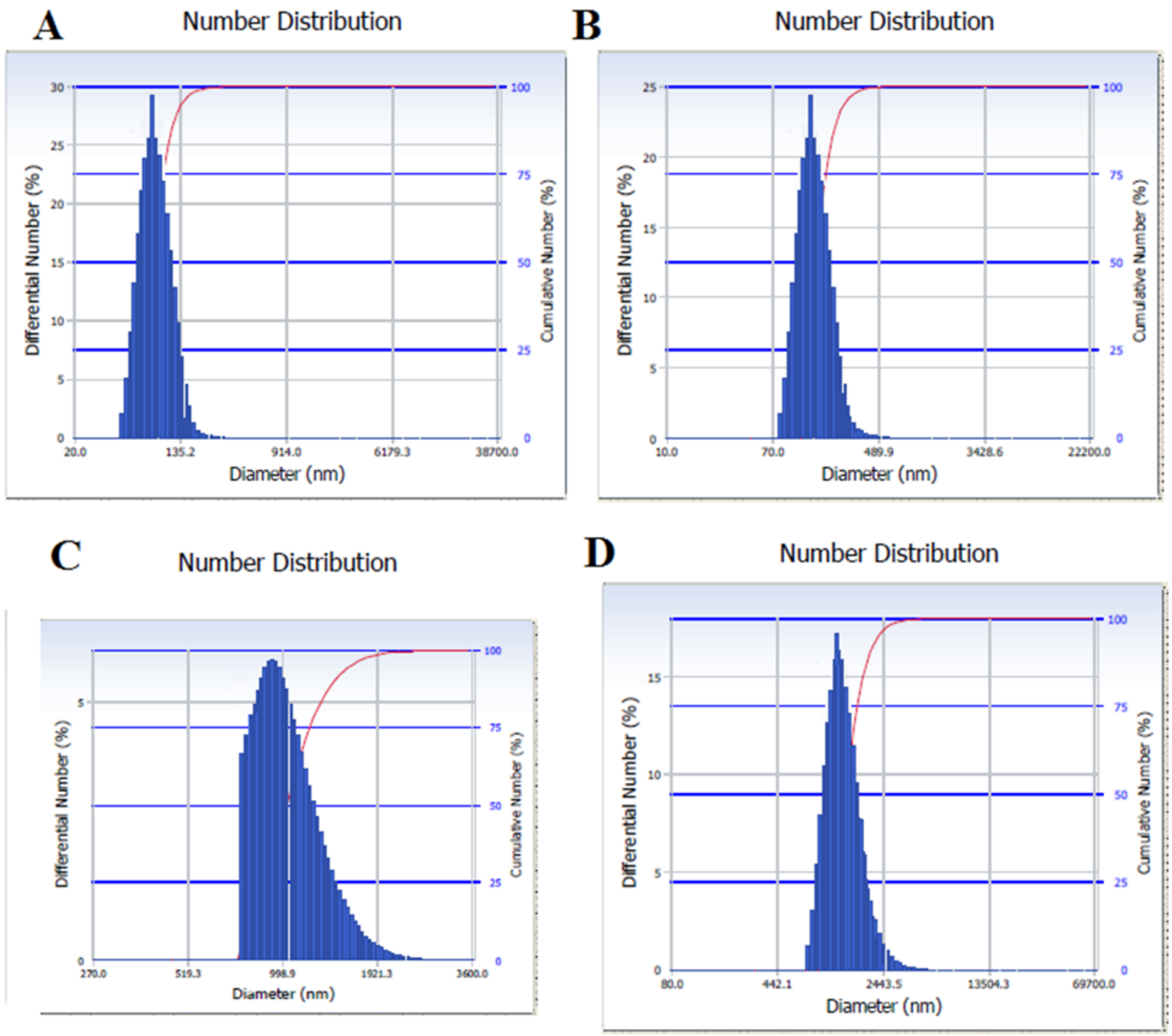

Figure S11: DLS analysis of the number distribution along the hydrodynamic radius of (A) S1/S2 DNA coassembly with Zn PpIX; (B) S1/S2 DNA–TPA diconjugate coassembly with Zn PpIX; (C) S1/S2 DNA–TPA triconjugate self-assemblies; (D) S1/S2 DNA–TPA triconjugate coassembly with Zn PpIX.

Atomic force microscopy (AFM):

Samples were prepared by drop casting 20 µL of annealed DNA solution in the presence and absence of Zn PpIX on freshly prepared APS mica. The AFM imaging was performed in intermittent contact mode atomic force microscopy by using an Agilent 5500 scanning probe microscope. Commercial silicon nitride cantilevers having a force constant of 1.2–5.5 N/m were used for the imaging of DNA nanostructure (MiKromash, Bulgaria). During imaging the cantilever was oscillating at its resonance frequency ranging from 60–90 kHz. The set point ratio of the cantilever, which govern the tapping forces, varied from 0.2 to 0.4.

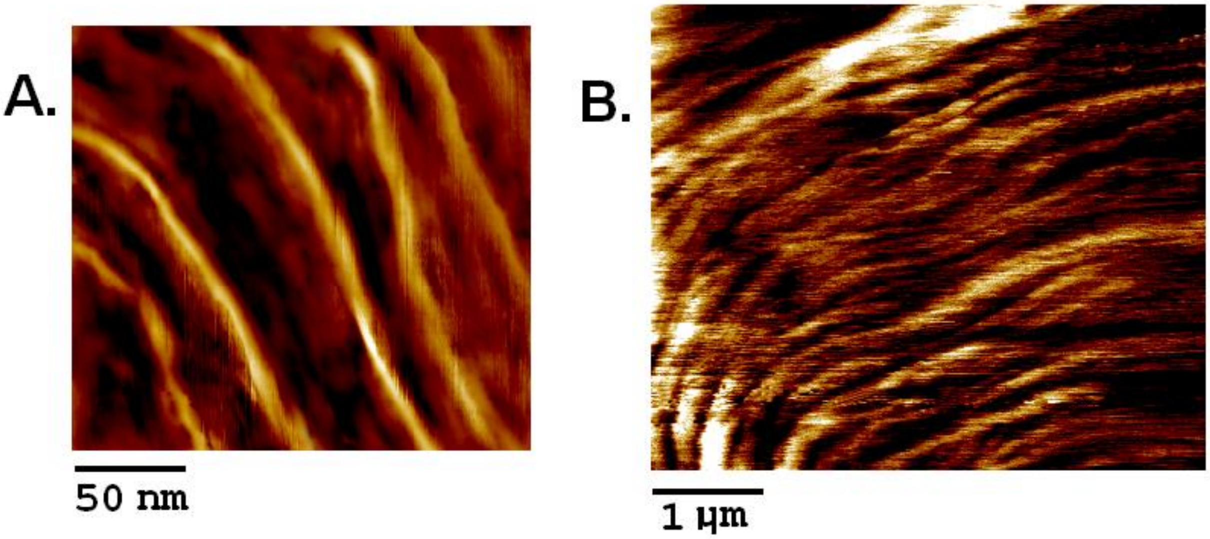

Figure S12: A. AFM images of DNA–TPA self-assemblies with Zn PpIX. B. Same as A at a different resolution

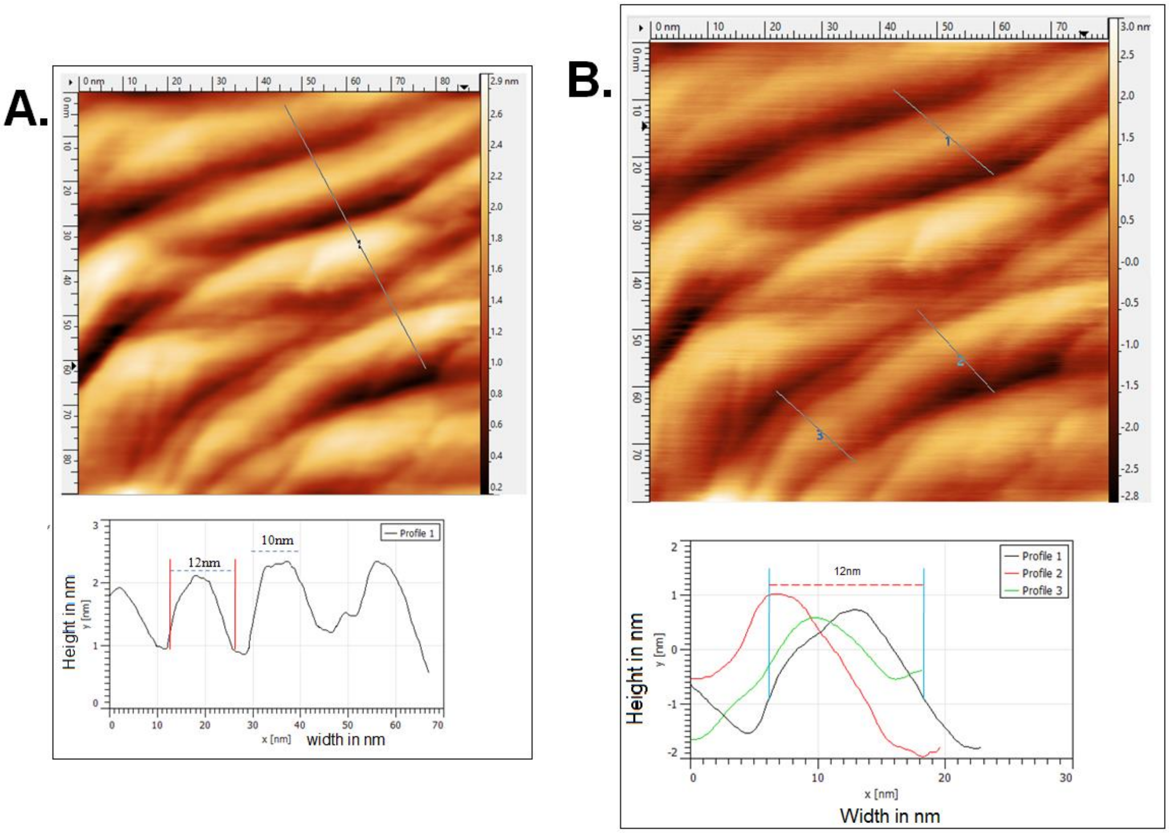

Figure S13. Height profile of AFM images of DNA–TPA triconjugate self-assembled in presence of Zn PpIX indicating height and width in ranges of 0.8–1.2 nm and 9–15 nm, respectively.

Modeling study of DNA–TPA single unit of nanofiber

Method: The carboxy acid derivative of triptycene, namely TPA was sketched using ChemDraw [1], which is a standard software tool for drawing the chemical molecules. The two dimensional structure of triptycene was then converted into a mol file. In order to generate the three-dimensional structure of TPA, the mol file was imported to the Schrodinger suite (Schrödinger, LLC, New York, NY, 2014) [2]. The 3D structure of TPA was then preprocessed, which involved assigning the bond orders and addition of missing hydrogens to the structure. The similar procedure was followed for constructing the processed 3D structure of ZPP molecule. The Maestro's Build panel was employed to create the 12-mer ssDNA with sequence S1, 5'-TCAGTCAACAGC-3'. To the 5'-end of the ssDNA the amino group, NH_2 $(CH_2)_{12}$ was added covalently. Then, the 5'-aminated ssDNA was covalently conjugated to all end groups of

TPA to form the S1 DNA–TPA tri-conjugated adduct. Correspondingly, the 12-mer 5’-aminated ssDNA with complementary sequence S2, 5’-AGTCAGTTGTCG-3’ was also sketched, which was covalently conjugated to all the ends of the TPA molecule to constitute the S2 DNA–TPA tri-conjugated adduct. Each of these TPA-conjugated structures associates to constitute the DNA nano-ladder.

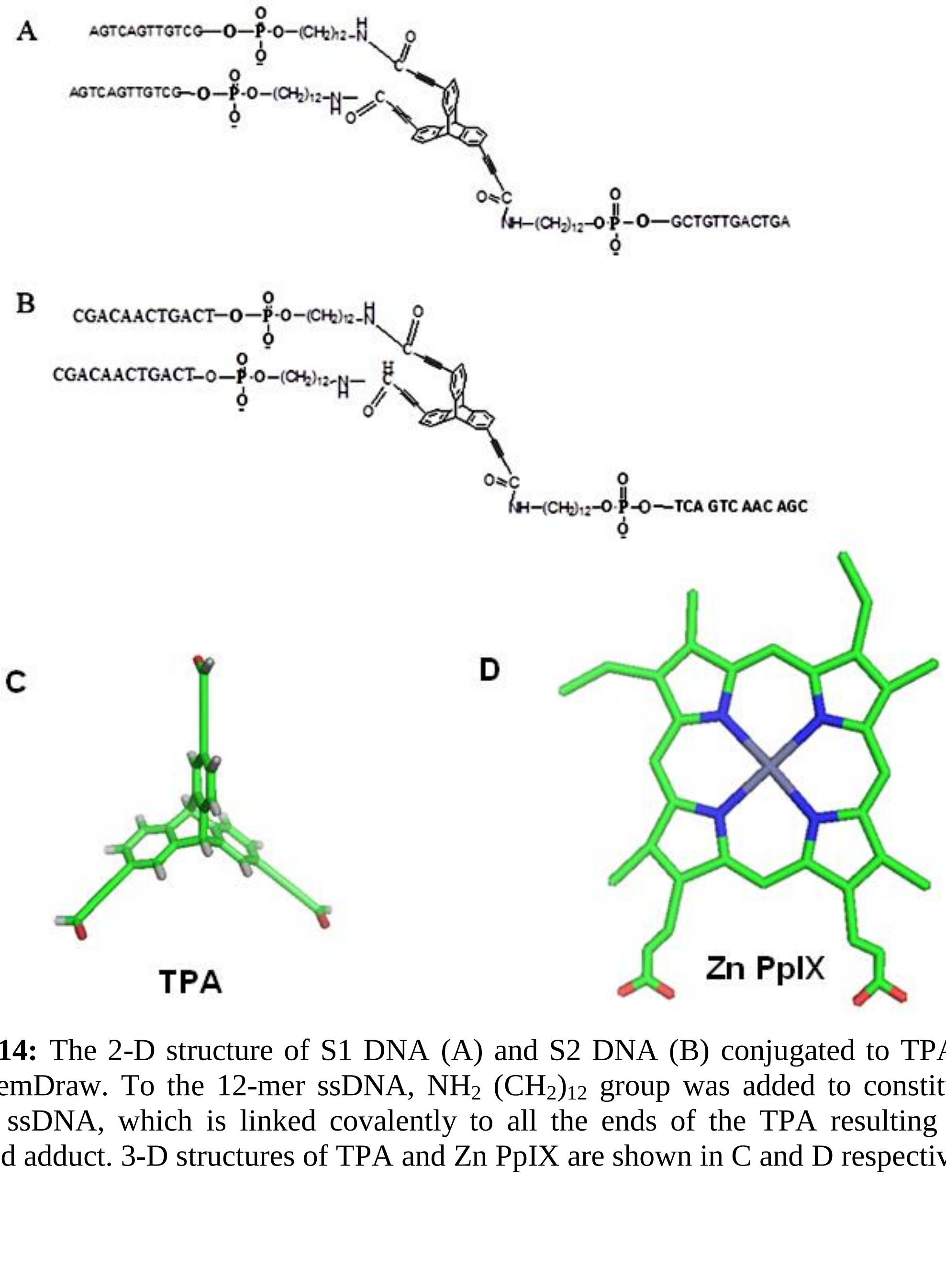

Figure S14: The 2-D structure of S1 DNA (A) and S2 DNA (B) conjugated to TPA sketched using ChemDraw. To the 12-mer ssDNA, NH_2 $(CH_2)_{12}$ group was added to constitute the 5′-aminated ssDNA, which is linked covalently to all the ends of the TPA resulting in the tri-conjugated adduct. 3-D structures of TPA and Zn PpIX are shown in C and D respectively.

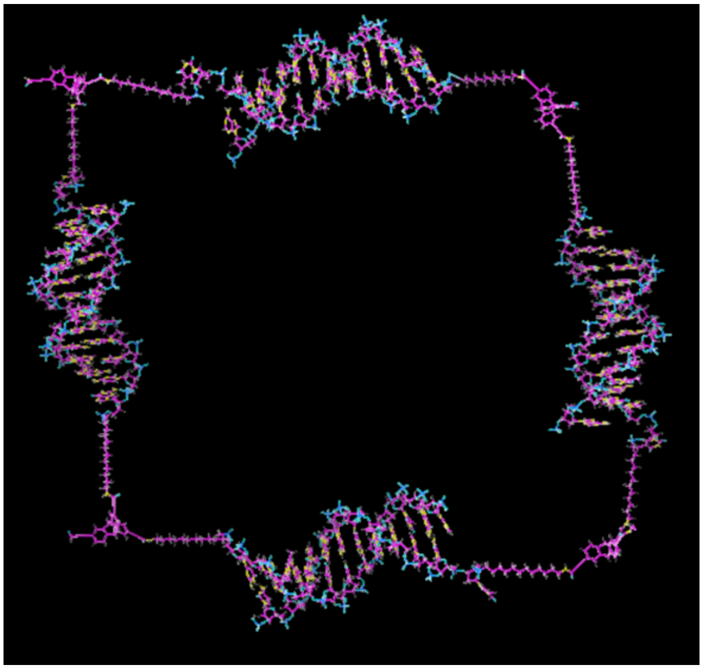

Figure S15: Modeling of self-assembly of S1–TPA and S2–TPA diconjugates showing a closed tetrameric structure.

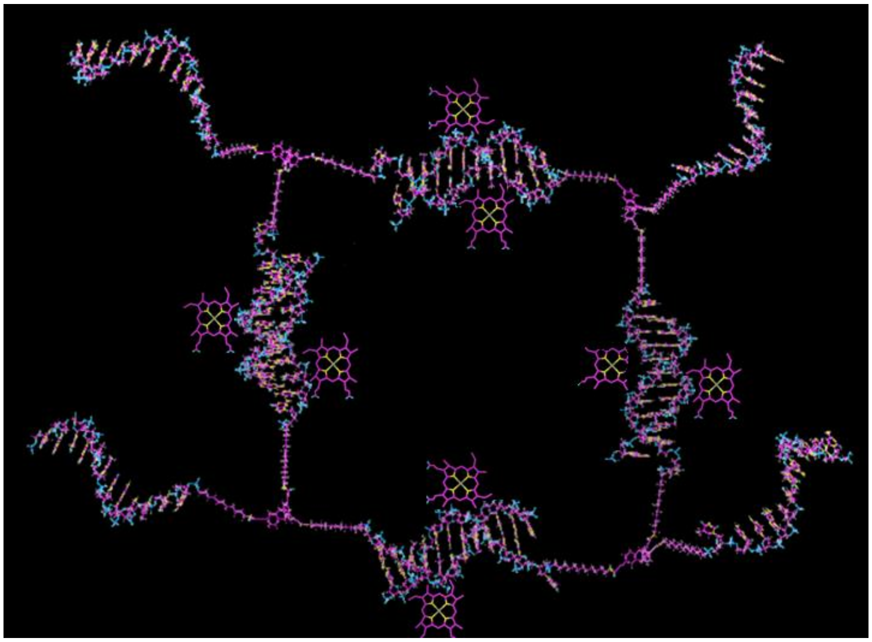

Figure S16: Modeling of the 3-D structure of S1–TPA/S2–TPA tetrameric units showing the interaction of Zn PpIX with DNA in a single building block of the nanofiber.

Circular dichroism

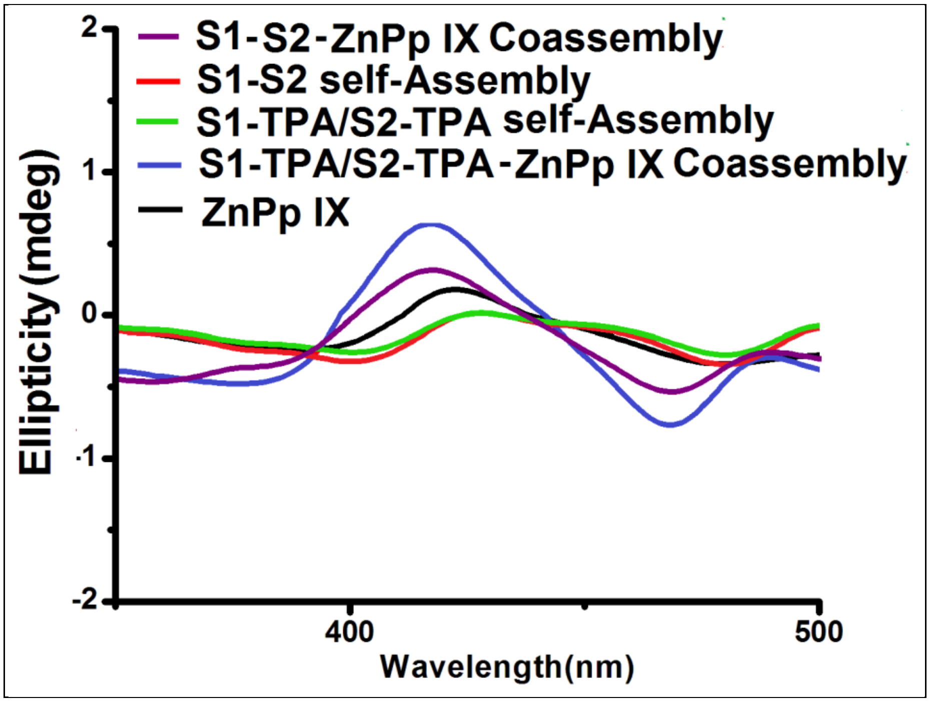

Figure S17: CD spectra showing interaction of Zn PpIX molecules with DNA–TPA triconjugates in the self-assembly and their controls.

References

1. Ultra, ChemDraw. "6.0 and Chem3D Ultra." Cambridge Soft Corporation, Cambridge, USA (2001).

2. Schrödinger Release 2014-3: Maestro, version 9.9, Schrödinger, LLC, New York, NY, 2014.
